# Sensitivity of portable low-field magnetic resonance imaging for multiple sclerosis lesions

**DOI:** 10.1016/j.nicl.2022.103101

**Published:** 2022-06-27

**Authors:** T. Campbell Arnold, Danni Tu, Serhat V. Okar, Govind Nair, Samantha By, Karan D. Kawatra, Timothy E. Robert-Fitzgerald, Lisa M. Desiderio, Matthew K. Schindler, Russell T. Shinohara, Daniel S. Reich, Joel M. Stein

**Affiliations:** a Department of Bioengineering, School of Engineering & Applied Science, University of Pennsylvania, Philadelphia, PA 19104, USA; b Center for Neuroengineering and Therapeutics, University of Pennsylvania, Philadelphia, PA 19104, USA; c Penn Statistics in Imaging and Visualization Center and Department of Biostatistics, Epidemiology, and Informatics, University of Pennsylvania, Philadelphia, PA 19104, USA; d National Institute of Neurological Disorders and Stroke, National Institutes of Health (NIH), Bethesda, MD 20892, USA; e Hyperfine, Guilford, CT 06437, USA; f Department of Radiology, Perelman School of Medicine, University of Pennsylvania, Philadelphia, PA 19104, USA; g Department of Neurology, Perelman School of Medicine, University of Pennsylvania, Philadelphia, PA 19104, USA

**Keywords:** Low-field MRI, Portable MRI, Point-of-care MRI, Hyperfine, Multiple sclerosis, White matter lesions

## Abstract

Magnetic resonance imaging (MRI) is a fundamental tool in the diagnosis and management of neurological diseases such as multiple sclerosis (MS). New portable, low-field strength, MRI scanners could potentially lower financial and technical barriers to neuroimaging and reach underserved or disabled populations, but the sensitivity of these devices for MS lesions is unknown. We sought to determine if white matter lesions can be detected on a portable 64mT scanner, compare automated lesion segmentations and total lesion volume between paired 3T and 64mT scans, identify features that contribute to lesion detection accuracy, and explore super-resolution imaging at low-field. In this prospective, cross-sectional study, same-day brain MRI (FLAIR, T1w, and T2w) scans were collected from 36 adults (32 women; mean age, 50 ± 14 years) with known or suspected MS using Siemens 3T (FLAIR: 1 mm isotropic, T1w: 1 mm isotropic, and T2w: 0.34–0.5 × 0.34–0.5 × 3–5 mm) and Hyperfine 64mT (FLAIR: 1.6 × 1.6 × 5 mm, T1w: 1.5 × 1.5 × 5 mm, and T2w: 1.5 × 1.5 × 5 mm) scanners at two centers. Images were reviewed by neuroradiologists. MS lesions were measured manually and segmented using an automated algorithm. Statistical analyses assessed accuracy and variability of segmentations across scanners and systematic scanner biases in automated volumetric measurements. Lesions were identified on 64mT scans in 94% (31/33) of patients with confirmed MS. The average smallest lesions manually detected were 5.7 ± 1.3 mm in maximum diameter at 64mT vs 2.1 ± 0.6 mm at 3T, approaching the spatial resolution of the respective scanner sequences (3T: 1 mm, 64mT: 5 mm slice thickness). Automated lesion volume estimates were highly correlated between 3T and 64mT scans (r = 0.89, p < 0.001). Bland-Altman analysis identified bias in 64mT segmentations (mean = 1.6 ml, standard error = 5.2 ml, limits of agreement = –19.0–15.9 ml), which over-estimated low lesion volume and under-estimated high volume (r = 0.74, p < 0.001). Visual inspection revealed over-segmentation was driven venous hyperintensities on 64mT T2-FLAIR. Lesion size drove segmentation accuracy, with 93% of lesions > 1.0 ml and all lesions > 1.5 ml being detected. Using multi-acquisition volume averaging, we were able to generate 1.6 mm isotropic images on the 64mT device. Overall, our results demonstrate that in established MS, a portable 64mT MRI scanner can identify white matter lesions, and that automated estimates of total lesion volume correlate with measurements from 3T scans.

## Highlights

• Paired, same-day, 3T and 64mT MRI studies were analyzed in 33 MS patients.
• 64mT MRI showed 94% sensitivity for detecting any lesions in 3T confirmed cases.
• The diameter of the smallest detected lesion was larger at 64mT compared to 3T.
• Total lesion volume estimates were strongly correlated between 3T and 64mT scans.
• Portable low-field MRI detects white matter lesions, but smaller lesions may be missed.

## Graphical abstract

**Figure:**
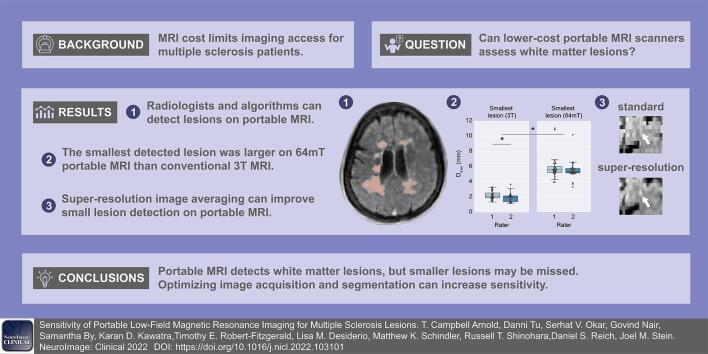

## 1 Introduction

Multiple sclerosis (MS) is a complex inflammatory and degenerative disease of the central nervous system (Longo et al., 2018). MS causes demyelinating lesions, typically assessed using magnetic resonance imaging (MRI). Imaging features related to white matter lesions (WMLs), such as number, volume, and dissemination in space and time, are key diagnostic criteria of MS (Thompson et al., 2018) and determine treatment courses and clinical trial eligibility (Filippi et al., 2019). Early diagnosis leads to better clinical outcomes, including delayed disease progression and reduced severity (Noyes and Weinstock-Guttman, 2013).

High-field strength MRI (1.5–3T) plays a crucial role in the diagnosis and management of MS. The 2021 consensus recommendations from the MAGNIMS, NAIMS, and CMSC working groups endorsed high-field MRI for MS diagnosis (Wattjes et al., 2021). 3T MRI is preferred over 1.5T because these scanners offer higher lesion detection rates and shorter acquisition times (Wattjes et al., 2021), although this has not been shown to lead to earlier diagnosis (Hagens et al., 2019, Hagens et al., 2018). Today, 1.5T devices remain the most common diagnostic MRI scanners in the United States. The working groups explicitly do not recommend using scanners with a field strength below 1.5T.

Although MS affects ∼ 800,000 people in the United States (Wallin et al., 2019) and likely > 2.5 million people globally (Tullman, 2013), the significant cost, infrastructure, and technical requirements associated with traditional high-field strength systems limits access to MRI worldwide (Marques et al., 2019). The scarcity is particularly felt in low-resource, sparsely populated, and rural areas (Ogbole et al., 2018). In some countries, the majority of MRI systems are low-field strength (<1T), while in others there are no available scanners at all (Ogbole et al., 2018, Geethanath and Vaughan, 2019). In the West African region, there are <100 MRI units serving a population larger than the United States, with a majority of the available devices being low-field strength (Ogbole et al., 2018, Jimeno et al., 2022). Portable, low-field MRI could play a significant role in providing imaging services in areas where even 1.5T scanners are out of financial reach. Even within the United States 60% of rural hospitals lack on-site MRI (Ginde et al., 2008). As the lack of diagnostic imaging can lead to delayed diagnosis and treatment, which result in worsening health disparities (Maru et al., 2010), there is renewed interest in low-field MRI as a lower-cost and potentially portable alternative to high-field MRI for neurologic disease generally (Wald et al., 2020).

Recent improvements in hardware as well as image reconstruction and processing algorithms (Campbell-Washburn et al., 2019) have made low-field MRI promising in contexts where modest resolution is sufficient for diagnostic purposes (Danni et al., 2020). The clinical utility of portable low-field MRI has been investigated for bedside monitoring in intensive care settings, where patients may not be stable enough to transport for traditional imaging (Sheth et al., 2021, Mazurek et al., 2021, Turpin et al., 2020). In the outpatient treatment of diseases such as MS, portable low-field MRI has the potential to increase access to MRI technology and enable more frequent monitoring of disease activity (Scarpazza et al., 2020). However, to achieve a diagnostically useful signal-to-noise ratio (SNR), low-field sequences typically require larger voxel sizes or longer scan times. It remains unknown to what extent lower image resolution and differences in tissue contrast associated with very low magnetic field strength will affect WML detection on these new portable devices.

In this study, we assessed the feasibility of portable low-field MRI for MS lesion identification and lesion volume estimation. We collected paired same-day 3T and 64mT brain MRI scans from adults with known or suspected MS at two different institutions. We used standard protocols (designed to meet MS consortium guidelines) for clinical Siemens 3T imaging and standard sequences (developed by Hyperfine to provide typical tissue contrasts for general brain imaging in reasonable resolution and scan time) at 64mT (Wattjes et al., 2021, Wattjes et al., 2015). We then compared lesion detection between scanners using both manual and automated measurements. We anticipated that tissue contrast would be sufficient to detect MS lesions at 64mT but sensitivity for small lesions would be reduced due to the lower resolution of the low field sequences. Finally, we explored a simple approach for super-resolution imaging of small lesions based on multi-acquisition image averaging.

## 2 Materials and methods

### 2.1 Participants & imaging

**Fig. 1:**
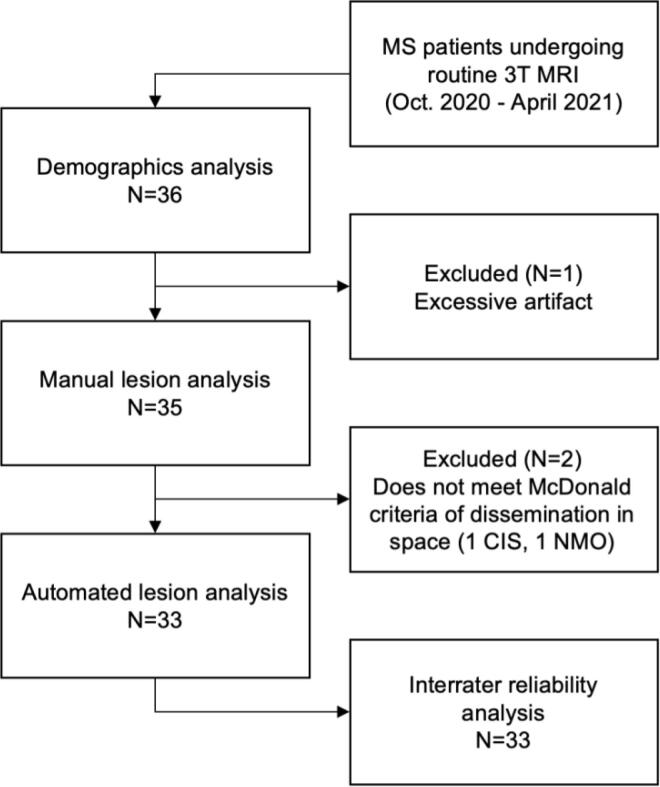
Flow chart of study participants. Abbreviations: multiple sclerosis (MS), clinically isolated syndrome (CIS), neuromyelitis optica (NMO).

Among adult outpatients undergoing clinical 3T brain MRI for known or suspected MS between October 2020 and April 2021, 36 patients (Fig. 1) were recruited at site A (N = 21) and site B (N = 15). All patients received same-day 3T and 64mT MRI. Demographic information was collected from clinical notes and included age, sex, race, clinical phenotype, disease duration, Expanded Disability Status Scale (EDSS), and current disease modifying therapy (Table 1). This study was approved by each site’s institutional review board, and patients provided written, informed consent.

**Table 1:** Patient Demographics. Demographic information and clinical history for 36 consecutive MS patients included in the study. An asterisk indicates a significant difference between sites. Abbreviations: Expanded disability status scale (EDSS), relapsing-remitting multiple sclerosis (RRMS), primary progressive multiple sclerosis (PPMS), secondary progressive multiple sclerosis (SPMS), clinically isolated syndrome (CIS), radiologically isolated syndrome (RIS), neuromyelitis optica (NMO), idiopathic transverse myelitis (ITM).

	Total (N = 36)	Site A (N = 21)	Site B (N = 15)
Age (years)	49.6 ± 14.2	45.3 ± 13.6 *	55.7 ± 12.7 *
Sex (women/men)	32/4	19/2	13/2
Race/ethnicity (White/Black/Hispanic)	26/9/1	14/7/0	12/2/1
Disease duration (years)	13.7 ± 11.2	10.2 ± 9.6 *	18.5 ± 11.5 *
EDSS (0–10)	1.5 (IQR = 2.0)	1.5 (IQR = 2.0)	2.0 (IQR = 1.25)
Phenotype		RRMS (18), CIS (1), NMO (1), RIS (1)	RRMS (10), SPMS (2), CIS (1), ITM (1), PPMS (1)
Current disease modifying therapy		ocrelizumab (9), natalizumab (2), other (6), none (4)	dimethyl fumarate (6), ocrelizumab (3), other (3), none (6)

**Fig. 2:**
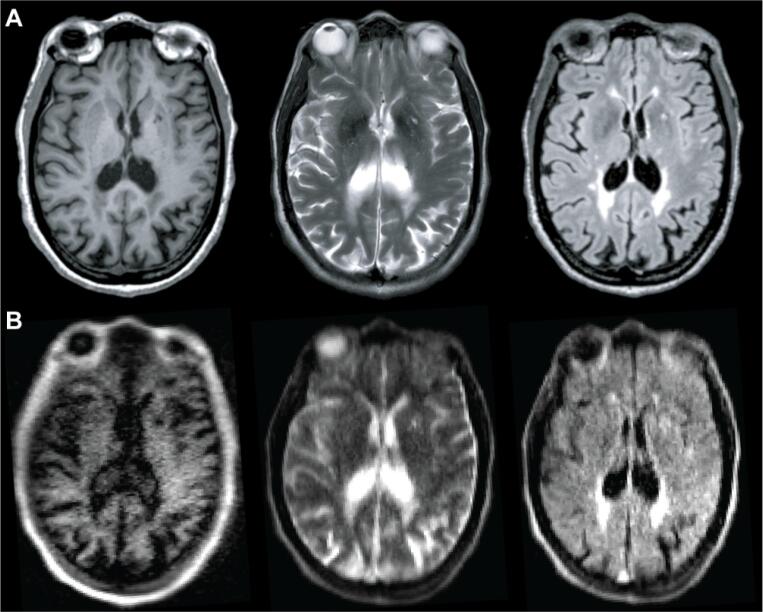
MS lesions on 3T and 64mT pulse sequences. Paired 3T (A) and 64mT (B) images from a 66-year-old woman with stable RRMS. Sequences include T1w (left), T2w (center), and T2-FLAIR (right). Images from both scanners show deep gray matter lesions and periventricular white matter lesions. Note also the superior sagittal sinus is hyperintense in the 64mT T2w and T2-FLAIR sequences, but not at 3T.

High-field MRI was performed on 3T scanners (Siemens, Erlangen, Germany). Each site used a standardized, whole-brain imaging protocol, which included 3D T1-weighted (T1w), T2-weighted (T2w), and 3D T2-FLAIR sequences (Fig. 2A). Sequence parameters are listed in Table 2. Total scan time was 11:06 min at site A and 20:15 min at site B. Patients at site B received gadolinium (gadobutrol, 0.1 mmol/L) prior to 3T scanning and 64mT scans were obtained after the contrast-enhanced 3T scans with mean post-gadolinium duration of 58 ± 21 min. Same-day, low-field MRI was performed on portable 64mT Swoop MRI systems (Hyperfine, Guilford, CT). Whole-brain 3D fast spin-echo T1w, T2w, and T2-FLAIR scans, analogous to those acquired at 3T, were collected (Fig. 2B). The 64mT imaging incorporates undersampling and deep-learning based reconstruction to increase SNR and decrease scan time. Total scan time was 21:22 min.

**Table 2:** Sequence parameters for study scans. Abbreviations: Tesla (T), T1-weighted (T1w), T2-weighted (T2w), Fluid-attenuated inversion recovery (FLAIR), echo time (TE), repetition time (TR), inversion time (TI), fast spin echo (FSE), magnetization-prepared rapid gradient echo (MPRAGE), magnetization-prepared 2 rapid gradient echo (MP2RAGE).

Sequence	Site	Field Strength (T)	TE (ms)	TR (s)	TI (s)	Resolution (mm)	Scan-time (min:sec)	Averages
T1w MPRAGE	A	3	2.48	1.9	0.9	1.0x1.0x1.0	4:18	1
T1w MP2RAGE	B	3	2.92	5	0.7, 2.5	1.0x1.0x1.0	8:30	1
T1w FSE	Both	0.064	6.26	1.5	0.3	1.5x1.5x5	4:52	1
T2-FLAIR	A	3	398	5	1.6	1.0x1.0x1.0	5:02	1
T2-FLAIR	B	3	352	4.8	1.8	1.0x1.0x1.0	7:15	1
T2-FLAIR FSE	Both	0.064	200	4	1.4	1.6x1.6x5	9:29	1
T2w	A	3	103	5.5	N/A	0.5x0.5x5.2	1:46	2
T2w	B	3	82	5	N/A	0.34x0.34x3.0	4:30	1
T2w FSE	Both	0.064	209	2	N/A	1.5x1.5x5	7:01	1

### 2.2 Manual review and lesion measurement

MRI scans were reviewed for WMLs by two neuroradiologists (DSR and JMS, 19 and 8 years of experience, respectively), and images with significant artifacts or were excluded from subsequent analysis. Maximum diameters (Dmax) of the smallest and largest WML visually detectable at each field strength were manually measured by a neuroradiologist (JMS) and a neurologist (SVO) with MS MRI expertise (3 years of experience) using ITK-SNAP (Yushkevich et al., 2006). All measurements were made on T2-FLAIR scans. Lesions were assessed in axial planes as well as sagittal and coronal reformatted images, and Dmax measurements were made on the plane with the largest lesion diameter. In confluent periventricular lesions, Dmax was measured perpendicular to the ventricle. Low-field imaging was evaluated prior to 3T scans to avoid interpretation bias, and image sets were reviewed separately. Inter-rater reliability was assessed using two-way random, single-measure intraclass correlation coefficients (ICC) with 95% confidence intervals (CI) reported. Patients who did not meet the McDonald criteria for dissemination of lesions in space were excluded from subsequent analyses (Thompson et al., 2018).

### 2.3 Quantifying image quality

Image quality was assessed using four quantitative metrics: Lesion conspicuity, SNR, contrast-to-noise-ratio (CNR), and variance of the Laplacian. Lesion conspicuity quantifies lesion intensity relative to normal appearing ipsilateral white matter. WMLs and normal appearing ipsilateral white matter regions were manually segmented on 3T and 64mT imaging using ITK-SNAP (Yushkevich et al., 2006). We calculated lesion conspicuity as the ratio of the difference and the sum of mean intensity in the two segmentations:

(1) $$\mathrm{Conspicuity}=\frac{\mu_{L}-\mu_{\mathrm{WM}}}{\mu_{L}+\mu_{\mathrm{WM}}},$$

where $\mu_{L}$ is the mean intensity in the lesion segmentation and $\mu_{\mathrm{WM}}$ is the mean intensity of the normal appearing ipsilateral white matter segmentation.

SNR and CNR are related measures that place mean lesion and white matter intensity in context to image noise. SNR compares the mean intensity of a signal to background noise, while CNR compares the contrast between two signals to background noise (Magnotta and Friedman, 2006). We calculated SNR and CNR as:

(2) $$\mathrm{SNR}=\frac{\mu_{L}}{\sigma_{\mathrm{AIR}}}$$

(3) $$\mathrm{CNR}=\frac{|\mu_{L}-\mu_{\mathrm{WM}}|}{\sigma_{\mathrm{AIR}}}$$

where $\sigma_{\mathrm{AIR}}$ is the standard deviation in a region of air surrounding the patient. Sampling air is meant to be representative of background noise in the images, however acquistion and reconstruction methods that impact the statisitical and spatial noise distribution can lead to over-or-underestimation of SNR. While only region-of-interest-based methods for SNR calculation were possible in this dataset, more robust difference measures should be evaluated in the future (Dietrich et al., 2007).

Variance of the Laplacian quantifies boundary sharpness and has been shown to be a robust measure of image focus (Pech-Pacheco et al., 2000, Pertuz et al., 2013). Variance of the Laplacian (ϕ) was calculated as:

(4) $$\phi_{x,y,k}=\;\overset{k+1}{\underset{f=k-1}{\sum }}\;\underset{\left(i,j\right)\in \Omega \left(x,y\right)}{\sum }\left|\Delta_{M}I_{f}\left(i,j\right)\right|$$

where $\Delta_{M}I_{f}$ is the Laplacian matrix of the image. Higher variance of the Laplacian corresponds to less image blurring. We registered each patient’s 3T image to the 64mT acquisition and calculated ϕ for both images to compare relative image blurring at low-field.

### 2.4 Automated lesion segmentation

The same WML segmentation pipeline was applied to 3T and 64mT images. Images were preprocessed using N4 bias correction (Tustison et al., 2010), and each T2-FLAIR volume was rigidly registered to the corresponding T1w volume using ﻿﻿Advanced Normalization Tools (ANTs) (Tustison et al., 2021, Avants et al., 2009). A brain mask was obtained using Multi-Atlas Skull-Stripping (MASS) (Doshi et al., 2013). To enable comparisons across patients, image intensities were normalized within each sequence using White Stripe (Shinohara et al., 2014). Lesion segmentation was performed using the Method for Inter-Modal Segmentation Analysis (MIMoSA) (Valcarcel et al., 2018, Valcarcel et al., 2018), an automated pipeline developed for 3T data that leverages shared information (coupling) between modalities to produce probability maps of WMLs (Fig. S1). To generate binary lesion masks all probability maps were thresholded at 0.2, a value manually selected based on prior empirical evidence.

### 2.5 Automated segmentation evaluation

Estimation of total lesion volume was the primary performance measure compared between 3T and 64mT segmentations. Two lesion volume estimates were obtained for each patient by calculating lesion segmentation volumes for the respective scanners. The relationship between volume estimates was assessed using Pearson’s correlation. Bland-Altman plots were used to determine agreement and assess for systematic scanner biases.

Similarity between segmentation masks was assessed using the Dice-Sørensen coefficient (Dice), which measures the overlap between two images (*X* and *Y*):

(5) $$\mathrm{Dice}=\frac{2\left|X\cap Y\right|}{\left|X\right|+\left|Y\right|}$$

Dice scores range from 0 to 1, with 1 indicating perfect segmentation overlap. Prior to Dice calculation, 3T and 64mT images were coregistered using ANTs (Avants et al., 2009). While Dice score may not reflect segmentation quality when the number of target objects is not known *a priori*, this measure was chosen as it is widely used and allows for comparisons across studies (Oguz et al., 2018). All 3T and 64mT segmentations were manually reviewed to verify overlapping regions were WMLs rather than false positive detections.

### 2.6 Size and intensity analysis

Connected-components analysis was used to identify individual lesions in automated 3T and 64mT segmentations (Boudraa et al., 2000). Sensitivity to individual lesions at low-field MRI was assessed using the true-positive rate (TPR), or the proportion of lesions correctly identified:

(6) $$\mathrm{TPR}=\frac{\mathrm{TP}}{\mathrm{TP}+FN}$$

where true positives (*TP*) are defined as lesions where 64mT and 3T segmentations overlap and false-negatives (*FN*) are defined as lesions with 3T segmentation but no 64mT segmentation overlap. The false-discovery rate (FDR) was assessed as:

(7) $$\mathrm{FDR}=\frac{\mathrm{FP}}{\mathrm{FP}+TP}$$

where false positives (*FP*) are defined as lesions with 64mT segmentation but no 3T segmentation overlap. Lesion overlap was defined as at least one shared voxel between the 3T and 64mT lesion segmentations. To understand the impact of lesion features on detection rates, TPR and FDR were plotted as a function of lesion size and normalized lesion intensity (Shinohara et al., 2014).

### 2.7 Super-resolution imaging

Low-field MRI particularly necessitates optimizing trade-offs between SNR, scan time and image resolution, which may limit the minimum detectable lesion size. However, image quality and resolution can be increased by taking advantage of signal averaging and partial volume effects in multiple scans (Jovicich et al., 2009, Deoni et al., 2022). In two patients and one control participant with WMLs, we explored multi-acquisition volume averaging techniques.

In the first patient, eight 64mT T2-FLAIR axial acquisitions (TE = 0.19 s, TR = 4 s, TI = 1.4 s, averages = 4, scan time = 6:03 min, resolution = 1.8x1.8x5 mm) were collected with head repositioning between scans (total scan time: 48:24 min). In addition to slightly lower resolution, these acquisitions used greater undersampling to achieve a shorter scan time. Super-resolution images were iteratively generated for each additional acquisition by reslicing images to 1.8 mm isotropic voxel sizes, affine registration to the initial acquisition, and averaging to create a higher-resolution volume. Lesion conspicuity of a small 0.06 ml lesion was quantified for each iteration.

In the second patient, three 64mT T2-FLAIR images were acquired in orthogonal slice-select directions (axial: TE = 0.19 s, TR = 4 s, TI = 1.4 s, averages = 4, scan time = 9:02 min, resolution = 1.6x1.6x5 mm, sagittal: TE = 0.23 s, TR = 4 s, TI = 1.4 s, averages = 4, scan time = 8:27 min, resolution = 5x1.6x1.6 mm, coronal: TE = 0.21 s, TR = 4 s, TI = 1.4 s, averages = 4, scan time = 8:10 min, resolution = 1.6x5x1.6 mm). Images were resliced to 1.6 mm isotropic voxel sizes, registered to the initial acquisition, and averaged to create a single high-resolution volume. Lesion conspicuity was calculated for each individual acquisition and the super-resolution images.

Imaging was also collected in a control participant with incidental nonspecific WMLs that are probably sequelae of chronic small vessel ischemia. In this case, we generated T1w, T2w, and T2-FLAIR super-resolution images from three sets of orthogonal acquisitions. The T2w and T2-FLAIR sequences again used greater undersampling to reduce scan time (T2w: axial = 2:53 min, sagittal = 1:59 min, coronal = 2:21 min, total scan time = 7:13 min; T2-FLAIR: axial = 6:03 min, sagittal = 5:02 min, coronal = 6:02 min, total scan time = 17:07 min). Full sequence parameters are available in supplementary Table S1.

### 2.8 Statistics and Data/Code availability

All code related to this study is publicly available. The MIMoSA algorithm is available in R as a Neuroconductor package and on GitHub (https://github.com/avalcarcel9/mimosa/). T-tests, Pearson’s correlation, and summary statistics were calculated using scipy (v1.5.2) and numpy (v1.19.2) in Python (v3.8.5). Bland-Altman plots were visualized using pyCompare (v1.5.1) while boxplots and correlations utilized seaborn (v0.11.0). Inter-rater reliability was calculated using irr (v0.84.1) in R (v4.0.3). A manuscript companion containing all analyses is available on GitHub (https://github.com/penn-cnt/Arnold_LF-MRI_MS). The data generated in this study can be made available, with protected health information removed, upon reasonable request to the corresponding author and with a data sharing agreement between institutions in place.

## 3 Results

### 3.1 Patient demographics

We collected data from 36 adults with known or suspected MS. The patient population had a mean age of 49.6 (SD: 14.2) years and was composed of 32 women and 4 men (Table 1). The mean duration of disease was 13.7 years (SD: 11.2), and patients had a median EDSS of 1.5 (interquartile range = 2). Patients from site B were significantly older than those from site A (two-sample *t*-test, t = 2.2, p = 0.03, site A: 45.3 years old, site B: 55.7 years old) and had a correspondingly longer duration of disease (two-sample *t*-test, t = 2.3, p = 0.03, site A: 10.2 years, site B: 18.5 years). Additional demographic information is provided in Table 1. After initial scan review by clinicians, three patients were excluded from further analysis: One patient had 64mT image artifacts suspected to be caused by a large nearby metal structure (Fig. S2) and two patients did not meet the MS diagnostic criteria of having lesion dissemination in space (DIS) (Thompson et al., 2018). All other 64mT and 3T images were deemed to be of sufficient quality and free from interpretation-limiting motion artifacts. All subsequent analyses are based on the remaining 33 patients (Fig. 1).

### 3.2 Manual measurements

**Fig. 3:**
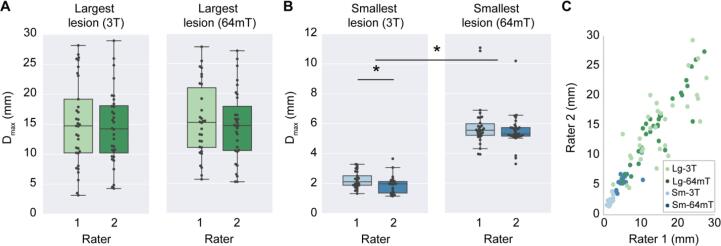
Manual lesion size measurements and interrater reliability. Raters from each site independently measured the maximum diameter (Dmax) of the smallest lesion (Sm) and largest lesion (Lg) in 3T and 64mT imaging for all patients. (A) For the largest lesion measurements, there was no significant difference between raters at 3T (t = 1.3, p = 0.19) or 64mT (t = 1.2, p = 0.23); additionally, there was no difference between 3T and 64mT measurements (t = 0.04, p = 0.97). (B) For the smallest lesion measurements, there was a significant difference between raters for 3T measurements (t = 4.83, p < 0.001) although 64mT measurements were not significantly different (t = 1.67, p = 0.11); additionally, the diameter of the smallest lesion was significantly lower (t = 19.6, p < 0.001) when measured on 3T (mean 2.1 mm) compared to 64mT (mean 5.7 mm). (C) Across all lesions there was a strong correlation (r = 0.90, p < 0.001) between raters. There was significant intraclass correlation for the largest lesion at 3T (ICC = 0.77, CI = [0.58–0.88]), largest lesion at 64mT (ICC = 0.91, CI = [0.83–0.96]), smallest lesion at 3T (ICC = 0.62, CI = [0.12–0.83]), and smallest lesion at 64mT (ICC = 0.66, CI = [0.4–0.82]), indicating a high degree of agreement between rater measurements for both 3T and 64mT.

MS lesions on 64mT are characterized by T1w hypointensity and T2w/T2-FLAIR hyperintensity, similar to 3T imaging (Fig. 2). At 64mT, lesions were identified by at least one rater in 94% (31/33) of patients with confirmed lesions on 3T imaging. In one patient, only one rater identified lesions at 64mT; all other low-field ratings were concordant. The largest and smallest lesions in each scan were identified, and the Dmax was recorded. The 64mT scanner showed 100% sensitivity for detecting WMLs when there was at least one lesion with Dmax > 5 mm (31/33 patients, 94%). Across patients, there was no significant difference in Dmax for the largest lesions measured at 64mT (15.1 ± 5.9 mm) and 3T (14.8 ± 6.6 mm) (Fig. 3A). However, the mean Dmax for the smallest detected WML was significantly larger (paired *t*-test, t = 19.6, p < 0.001) on 64mT (5.7 ± 1.3 mm) compared to 3T (2.1 ± 0.6 mm) (Fig. 3B). There was no effect of scan site on Dmax measurements, however there was a significant difference between rater 1 (2.3 ± 0.5 mm) and rater 2 (1.9 ± 0.6 mm) for the smallest lesion detected at 3T (paired *t*-test, t = 4.8, p < 0.001). No gadolinium enhancing WMLs were seen on 3T or 64mT imaging.

### 3.3 Interrater reliability

The smallest and largest lesion in each scan were independently measured by two raters to assess interrater reliability (Fig. 3). The ICC for each patient’s largest lesion measured at 3T and 64mT was 0.77 (CI = [0.58–0.88]) and 0.91 (CI = [0.83–0.96]) respectively, indicating high interrater reliability for large lesions on both scanners (Fig. 3A). Similarly, when measuring each patient’s smallest lesion there was a significant relationship between raters at both 3T (ICC = 0.62, CI = [0.12–0.83]) and 64mT (ICC = 0.66, CI = [0.4–0.82]) (Fig. 3B). This indicates that measurements made by raters had a similar degree of reliability at 3T and 64mT. Of note, the average smallest lesions detected (3T: 2.1 ± 0.6 mm, 64mT: 5.7 ± 1.3 mm) approached the slice thickness for the respective sequences (3T: 1 mm, 64mT: 5 mm).

### 3.4 Quantifying image quality

**Fig. 4:**
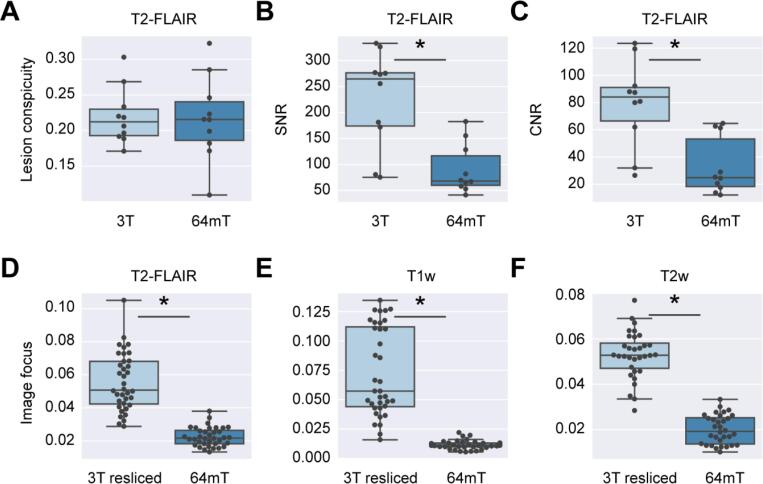
Quantitative comparison of lesion visibility and image blurring at 3T and 64mT. (A) Lesion conspicuity measures the intensity of a lesion relative to background tissue. Lesion conspicuity for the largest lesion was measured on 3T (light blue) and 64mT (dark blue) T2-FLAIR images for 10 patients. There was no significant difference in conspicuity between the scanners (paired *t*-test, t = 0.14, p = 0.89). (B) SNR compares mean lesion intensity to background noise. SNR was significantly higher in 3T images in the subset of 10 patients (paired *t*-test, t = 4.36, p = 0.00184). (C) Similarly, CNR, which compares the contrast between lesion and white matter to background noise, was also significantly higher in 3T imaging (paired *t*-test, t = 4.89, p < 0.001). (D-F) The variance of the Laplacian is a measure of image focus, with larger values indicating clearer images. We registered and resliced 3T to 64mT images and calculated this focus feature for both images. This process was carried out for (D) T2-FLAIR, (E) T1w, and (F) T2w sequences. Four subjects without T2w sequence pairs were excluded from panel D. For all sequences, low-field images were significantly more blurred than their resliced 3T counterparts (paired t-tests, T2-FLAIR: t = 11.6, p < 0.001, T1w: t = 9.5, p < 0.001, T2w: t = 19.5, p < 0.001). (For interpretation of the references to colour in this figure legend, the reader is referred to the web version of this article.)

To assess low-field image quality, we calculated lesion conspicuity, SNR, and CNR to quantify lesion visibility and the variance of the Laplacian to quantify image blurring. In an analysis run on a subset of 10 patients, we found lesion conspicuity was preserved in the T2-FLAIR low-field images (Fig. 4A, paired *t*-test, t = 0.14, p = 0.89). However, we found that SNR and CNR, which account for background noise, were both significantly lower in 64mT imaging (SNR: Fig. 4B, paired *t*-test, t = 4.36, p = 0.00184, CNR: Fig. 4C, paired *t*-test, t = 4.89, p < 0.001). This suggests there is similar lesion to tissue contrast between the low-field and high-field sequences, but more noise in low-field images. When assessing the relative focus of paired images across all subjects, we found low-field imaging was significantly more blurred than 3T images that were registered and resliced to match 64mT resolution (Fig. 4B-D). This effect was seen in T2-FLAIR (paired *t*-test, t = 11.6, p < 0.001), T1w (paired *t*-test, t = 9.5, p < 0.001), and T2w (paired *t*-test, t = 19.5, p < 0.001) acquisitions. Interrelated potential causes of blurring at low field include smaller matrix size/lower resolution, undersampling, patient motion, and high echo train length.

### 3.5 Total lesion volume estimates

**Fig. 5:**
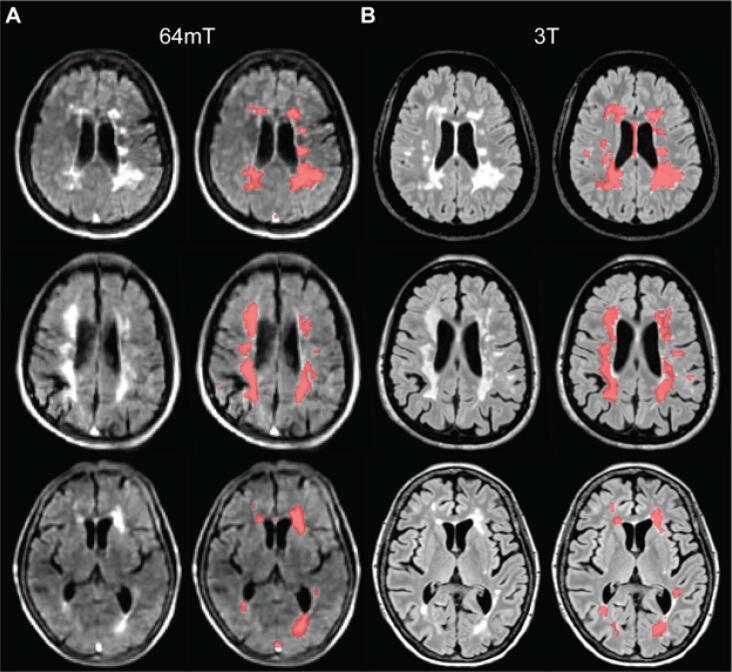
Automated lesion segmentations at 3T and 64mT overlap. (A) 64mT FLAIR images for three cases (left) with automated lesion segmentations generated from the 64mT images using MIMoSA overlaid (right). (B) Corresponding 3T FLAIR images for the same three cases (left) with 3T based segmentations (right). Patients from top to bottom are a 51-year-old female with RRMS, 44-year-old female with RRMS, and 71-year-old female with RRMS. All images were coregistered to 64mT T1-weighted images for comparison. Segmentations generated from 64mT and 3T scanners show similar patterns, although examples of false-positive segmentation in the sagittal sinus at 64mT can be seen in the top and bottom patients.

**Fig. 6:**
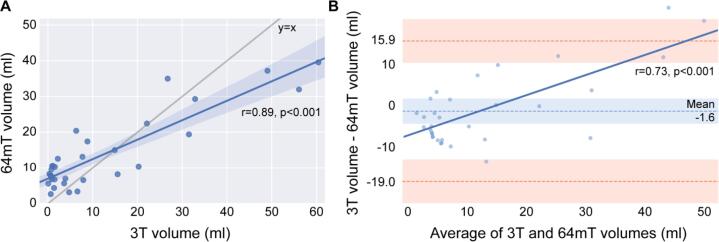
Total lesion volume measured at 3T and 64mT shows agreement. (A) 3T and 64mT total lesion volume estimates were strongly correlated (Pearson’s correlation, r = 0.89, p < 0.001). However, when compared to y = x there is a clear bias towards over-segmentation at low levels of total lesion volume. (B) A Bland-Altman plot illustrates the level of agreement between 3T and 64mT segmentation volumes (bias −1.6 ml, standard error of measurement = 5.2 ml, 95% limit of agreement −19.0 to 15.9 ml). The Pearson’s correlation (r = 0.74, p < 0.001) in dark blue further indicates over-segmentation at 64mT when lesion volume is low and under-segmentation when lesion volume is high. (For interpretation of the references to colour in this figure legend, the reader is referred to the web version of this article.)

To obtain more objective measures of lesion detection, 3T and 64mT image sets were processed with an automated lesion segmentation algorithm. Initial qualitative review of segmentation overlays revealed similar patterns of lesion segmentation, particularly with respect to large periventricular lesions (Fig. 5). Quantitative comparisons indicated that estimates of total lesion volume were highly correlated (r = 0.89, p < 0.001) (Fig. 6A). Mean lesion volume estimates were not significantly different (paired-*t*-test, t = 1.0, p = 0.32) between 3T (11.9 ± 16.5 ml) and 64mT (13.5 ± 10.2 ml) images. Upon visual inspection, however, some discrepancies were noted, such as significant false-positive detections on 64mT imaging in veins and peripheral cortical/subcortical artifacts (Fig. S3).

A Bland-Altman plot for agreement between 3T and 64mT lesion volume estimates is presented in Fig. 6B. The mean difference was 1.6 ml with a 5.2 ml standard error of measurement, and the 95% limits of agreement were −19.0 to 15.9 ml. There was a significant correlation (r = 0.74, p < 0.001) between pairwise differences and averages, indicating that compared to 3T, the 64mT segmentations overestimate low lesion volumes and underestimate high lesion volumes. Visual inspection revealed that false-positives contributing to over-segmentation were predominantly due to flow-related high signal intensity in veins, hyperintensity in non-lesion structures (such as the pineal gland), and areas of artifactual peripheral high signal in cortical/subcortical tissue on 64mT FLAIR sequences (Fig. S3).

### 3.6 Automated segmentation overlap

Across patients, there was a wide range in overlap between 3T and 64mT segmentation pairs (Dice: mean = 0.23, standard deviation = 0.21, max = 0.65, min = 0), with automated segmentations overlapping in 91% (30/33) of patients. Potential factors contributing to the overall low Dice score include false-positives and false-negatives on 64mT or 3T segmentations, registration errors between 64mT and 3T imaging, and differences in image resolution. Two patients had no segmentation overlap and one patient was excluded because the overlapping region was a hyperintense pineal gland, not a WML (Fig. S3 panel D). All three patients without lesion overlap were in the bottom 12% of total lesion volume, indicating algorithm performance may be poor for subjects with low lesion burden. Larger lesion size is frequently associated with higher Dice scores (Oguz et al., 2018). We found in our dataset that total lesion volume at 3T was highly correlated with Dice scores (r = 0.81, p < 0.001) (Fig. S4). Taken together, these results indicate that lesion segmentation did not perform as well when patients had a low lesion burden. To characterize the full range of segmentation quality across the dataset, Fig. S5 illustrates segmentations from each quartile of the Dice distribution.

### 3.7 Lesion sensitivity and false discovery

**Fig. 7:**
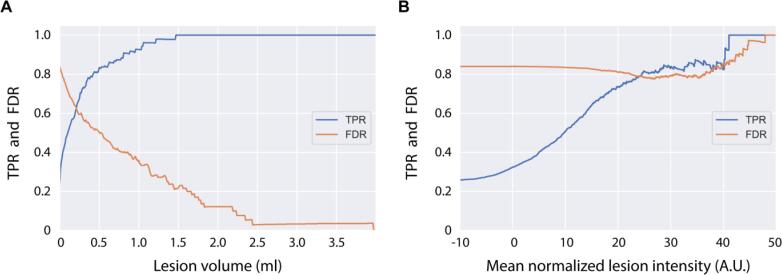
Lesion size and intensity influence detection rate. (A) The detection rate, or true positive rate (TPR), steadily increases with lesion size, with 93% detected at > 1 ml, and all lesions >1.5 ml being detected. The false discovery rate (FDR) decreases with lesion size, with 36% false discovery rate at > 1 ml, 22% at > 1.5 ml, and 3% at > 2.5 ml. Though the x-axis was limited to 4 ml for illustrative purposes, lesions > 20 ml were found in the dataset. (B) To analyze the relationship between lesion intensity and detection rate, image intensity values were first normalized using White Stripe (Shinohara et al., 2014). While detection rate increases as mean lesion intensity increases, the FDR remains high (>75%) across lesion intensities. The high number of false positive detections was driven by hyperintense veins and peripheral signal artifacts, as seen in Fig. S3.

In each segmentation, individual lesions were identified using connected-components analysis (Boudraa et al., 2000). For each lesion, volume and mean intensity were quantified. The true-positive rate (TPR) and false-discovery rate (FDR) were calculated across a range of lesion size and intensity thresholds (Fig. 7). The TPR increases dramatically with lesion size, reaching 93% for lesions > 1 ml and 100% for lesions > 1.5 ml. The FDR decreases with lesion size, reaching 36% for lesions > 1 ml, 22% for lesions > 1.5 ml, and 3% for lesions > 2.5 ml. TPR also increases with mean lesion intensity, indicating that lesion intensity influences sensitivity; however, FDR remains high (>75%) indicating a large number of false positive detections across intensity thresholds. Examples of false positive detections can be seen in Fig. S3.

### 3.8 Super-resolution imaging

**Fig. 8:**
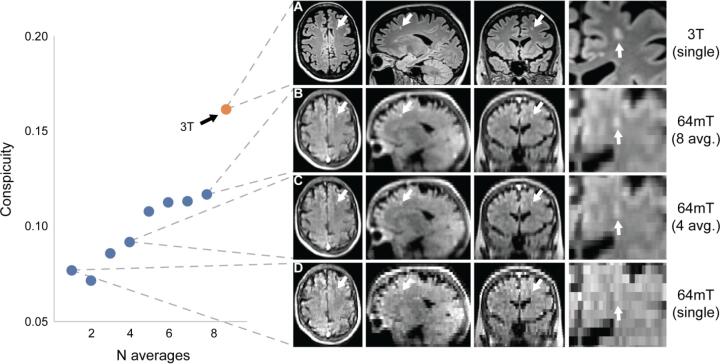
Multi acquisition image averaging can increase lesion conspicuity and resolution. This figure depicts a 3x4x5 mm (0.06 ml) subcortical left frontal white matter lesion in a 53-year-old woman with stable RRMS and compares 64mT FLAIR images generated from multi-acquisition image averaging to 3T imaging. The lesion is readily apparent on 3T imaging (A); however, it could not be discerned in a single 64mT acquisition (D). Volume averaging of multiple acquisitions with repositioning between scans did reveal the lesion on the low-field system (B & C). The lesion was discernible for N ≥ 3 multi acquisition averages. The lesion was manually segmented on 3T, and the ratio of mean lesion intensity to ipsilateral adjacent white matter (WM) is given as an estimate of lesion conspicuity (red dot). In 64mT imaging, the ratio steadily increases with additional acquisition averages (blue dots). With 8 vol averages, there was a 53% increase in lesion conspicuity. (For interpretation of the references to colour in this figure legend, the reader is referred to the web version of this article.)

We explored super-resolution imaging approaches using two additional patients and one control participant. In one patient, a 3x4x5 mm (0.06 ml) subcortical lesion was evident near the left middle frontal gyrus on 3T (Fig. 8A) but not in a single axial low-field acquisition (Fig. 8D). After multi-acquisition volume averaging of 3 to 8 acquisitions, the lesion became detectable on the low-field system, and lesion intensity relative to ipsilateral white matter steadily increased with additional acquisitions. With 8 vol averages, there was a 53% increase in lesion conspicuity, which was equivalent to 72% of conspicuity at 3T (Fig. 8B).

**Fig. 9:**
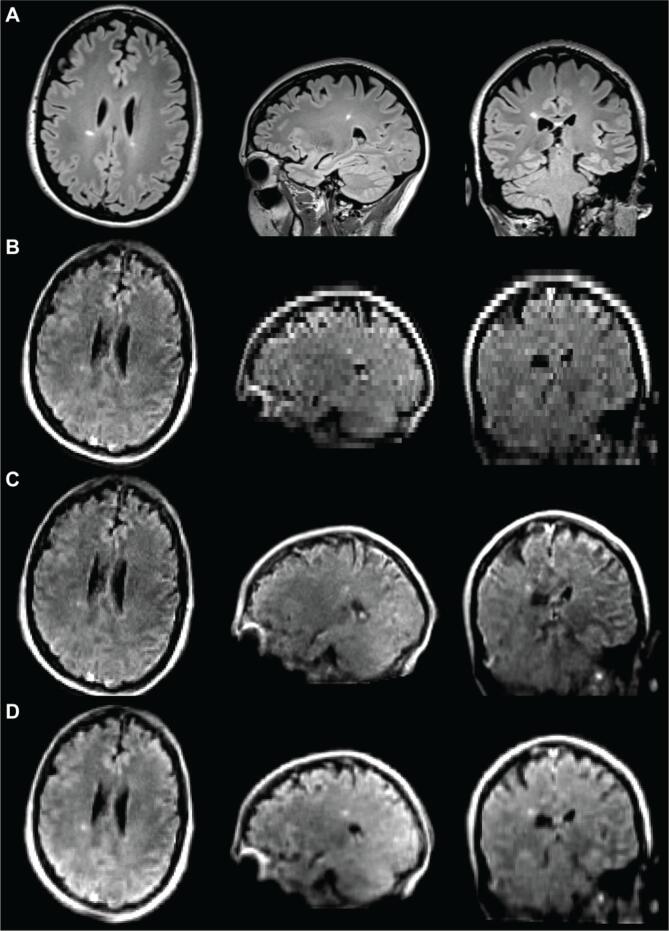
Super-resolution images generated from orthogonal slice directions. This figure demonstrates a super-resolution approach using anisotropic image acquired in orthogonal slice directions (axial, sagittal, and coronal). The patient is a 69-year-old man with stable RRMS. (A) 3T FLAIR imaging demonstrates a right periventricular lesion (conspicuity = 0.18) in the three orthogonal planes. (B) 64mT imaging of the same lesion (conspicuity = 0.11) using an axial FLAIR acquisition with sagittal and coronal reformatted images. (C) Corresponding axial, sagittal, and coronal slices from separate acquisitions in each direction (conspicuity: axial = 0.11, sagittal = 0.09, and coronal = 0.13) registered to the axial image by affine transformation. (D) Corresponding super-resolution images generated by averaging the coregistered axial, sagittal, and coronal acquisitions in C (conspicuity: linear = 0.11, nearest-neighbor = 0.13).

In the second patient, a 0.12 ml right periventricular lesion could be seen at 3T (Fig. 9A) and in a single axial low-field acquisition (Fig. 9B). The lesion could be better appreciated by aligning separate axial, sagittal, and coronal acquisitions (Fig. 9C). After multi-acquisition volume averaging of 3 orthogonal acquisitions, the lesion in the super-resolution image had a similar appearance to the coregistered axial, sagittal, and coronal acquisitions (Fig. 9D). Lesion conspicuity was higher in the 3T image (0.18) compared to the 3 orthogonal acquisitions (axial = 0.11, sagittal = 0.09, coronal = 0.13). Super-resolution image conspicuity was 0.11 with linear interpolation and 0.13 with nearest-neighbor interpolation, comparable to the original acquisitions. Although conspicuity was not higher in the super-resolution image for this example, resolution was increased to 1.6 mm isotropic, facilitating multi-planar review in a single volume.

In the control participant with incidental WMLs, we generated isotropic T1w, T2w, and T2-FLAIR super-resolution images (supplemental Fig. S7-9) using multi-acquisition volume averaging of three orthogonal acquisitions of each sequence (T1w = 16:50 min, T2w = 7:13 min, T2-FLAIR = 17:07 min, total scan time = 41:10 min, full scan parameters in supplementary Table S1). The longest individual acquisition was 6:03 min, making collection of these images more clinically feasible.

## 4 Discussion

In this study, we compared manual and automated lesion detection in paired 3T and portable 64mT brain MRI scans from patients with MS at two sites. On visual inspection of 64mT images, neuroradiologists were able to detect white matter lesions in 94% (31/33) of patients with discernable 3T lesions. An automated lesion segmentation algorithm detected overlapping lesions in 91% (30/33) of patients, and estimates of total lesion volume were highly correlated between 3T and 64mT scans (r = 0.89, p < 0.001). We investigated effects of lesion size on manual and automated detection, causes of false positive automated detections, and super-resolution imaging to increase resolution and lesion conspicuity at low-field. Our results suggest that portable 64mT imaging could have diagnostic utility in the context of MS, but further optimization and testing are needed.

Our efforts are motivated by recent advances in hardware development and reconstruction software that address the reduced SNR and resolution associated with low-field MRI. Though the earliest MRI scanners were low field strength, commercial systems have predominantly trended towards higher field strengths, with low-field systems relegated to niche applications (Marques et al., 2019, Campbell-Washburn et al., 2019, O’Reilly et al., 2021). Currently, there is renewed commercial interest in developing low-field MRI systems (Sarracanie et al., 2020), including Hyperfine’s portable 64mT Swoop system, Synaptive Medical’s 0.5T Evry system, and Siemens’s 0.55T Magnetom Free.Max system, all of which have received FDA clearance since 2020. As older and more recent literature indicate that very low-field systems can detect relatively subtle brain pathologies, including demyelinating disease (Orrison et al., 1991, Mateen et al., 2021, Arnold et al., 2022), we sought to investigate the sensitivity of the FDA-cleared portable 64mT MRI for MS lesions.

We found that clinicians could identify lesions on 64mT scans in 94% of patients with discernible lesions at 3T. Not surprisingly, the smallest detected lesion size was significantly larger at 64mT (5.7 ± 1.3 mm) compared to 3T (2.1 ± 0.6 mm). Lesion conspicuity depends not only on the inherent contrast between a lesion and surrounding tissue but also on the relative size or volume of the lesion with respect to the imaging resolution. Thus, imaging parameters should be optimized to maximize lesion-to-background contrast, although this can be challenging as MS lesions are known to have varying levels of myelination and tissue composition. Alternatively, one can improve the imaging resolution to reduce partial volume effects for better visualization of small lesions. Even though our 64mT sequences were not tailored for MS WML detection and might be optimized further, we found the lesion-to-background contrast of larger lesions to be comparable to 3T. Spatial resolution seemed to be the limiting factor for detecting small lesions, as the average voxel volume of the FLAIR images is about 13 times larger at 64mT than at 3T. Such low resolutions are used to compensate for the loss of SNR, which scales inversely with field strength and particularly accentuates the trade-off between resolution and scan time at low field. We used general purpose 64mT protocols developed by the scanner manufacturer to provide reasonable SNR and resolution with scan times for each sequence of <10 min.

Importantly, resolution can be increased through longer scan times or more acquisitions with averaging. We demonstrate this in our work combining multiple axial acquisitions, where we were able to resolve a lesion that was not evident on individual scans. The current limitations on clinical scan time are workflow constraints and patient motion and discomfort. However, the lower operational costs and decreased claustrophobia of some lower field strength devices may make longer scan sessions feasible. Advantages of acquiring multiple acquisitions instead of a single long acquisition include that information is obtained from each separate scan, any individual motion-degraded scan can be discarded, and scanning can continue as needed, tolerated, or to fill the time allowed. In addition, repositioning or a rotating field of view can take advantage of partial volume to increase resolution. An elegant version of this approach that we also illustrate combines orthogonal anisotropic images for super-resolution scans (Deoni et al., 2022). This strategy also has the advantage that individual MS lesions may be cross referenced between the original separate acquisitions or better seen in one plane or another due to their orientation, for example lesions perpendicular to the ventricular margin. Finding the right balance of resolution, sampling strategy, averaging, orientation and acquisition time will be an important goal of future work.

Whether gadolinium can be used to assess contrast-enhancing lesions on the 64mT device remains unknown. In our study cohort, we saw no contrast-enhancing lesions at 3T or 64mT, and contrast was not separately administered for low field scans. At lower field strengths, already short tissue T1-relaxation times reduce the benefit from T1 shortening contrast agents. A higher gadolinium dose (Desai and Runge, 2003), alternative higher relaxivity contrast agents (Desai and Runge, 2003, Bendszus et al., 2020), optimization of post-injection scan timing, and further pulse sequence optimization for contrast may be useful for low-field MRI. However, even at higher doses, low-field devices may have reduced sensitivity to contrast-enhancing lesions (Ertl-Wagner et al., 2001).

With clear advantages in resolution and scan time, gadolinium sensitivity, and brain and spinal cord imaging, high field MRI should at present remain the tool of choice for confirming and following MS. Where high field MRI is available, low field MRI should be viewed as complementary and not a replacement. Given its lower cost and ease of use, the most promising role for portable MRI in this context may be follow-up point-of-care imaging in established or suspected cases, where confirmatory high field imaging can be obtained when needed. In clinically isolated syndrome, earlier or more frequent portable MRI could support an MS diagnosis if able to show dissemination in time (Rovira et al., 2009). For established MS, portable MRI could permit earlier or more frequent imaging either for clinical changes, assessment of therapy response, or detection of treatment complications, such as progressive multifocal leukoencephalopathy (Scarpazza et al., 2020). Importantly, unenhanced imaging alone captures virtually all progressive disease on 3T MRI (Mattay et al., 2018, Sadigh et al., 2019, Eichinger et al., 2019). Though this is facilitated by high resolution and automated subtraction methods at 3T, if 64mT imaging can detect one or more new lesions or lesions of a threshold size felt to be clinically significant, then potentially lower sensitivity to gadolinium will be less of a detriment. How exactly to integrate low-field MRI into the longitudinal follow-up of MS patients requires additional research. Further studies should assess low-field MRI sensitivity for new or growing lesions over time.

Lower costs and infrastructure requirements of portable low-field MRI could expand clinical options for patients in low and middle income countries and rural areas with little access to MRI (Maru et al., 2010). Diagnostic utility of portable MRI in such settings will have to address questions of sensitivity, disease prevalence, and access to therapeutics. Patients with traditional relative MRI contraindications, including metal implants, pacemakers, and claustrophobia, may also benefit from reduced device interactions and open design at lower field strengths (Klein, 2016). While this will require additional safety determinations, recent work demonstrates that ﻿pacemaker leads and metallic guidewires can be safely imaged at 0.55T with minimal radiofrequency-induced heating (Campbell-Washburn et al., 2019). Additionally, most MS patients will experience mobility impairment, which can impact quality of care (Sutliff, 2010). Mobile MRI units could bring imaging to patients, providing otherwise unavailable service to sparsely populated areas and individuals who cannot travel (Shen et al., 2021, Deoni et al., 2022, Deoni et al., 2021).

Low-field MRI also offers the potential to conduct large-scale studies or screening of high-risk individuals at lower cost. In MS, high-risk asymptomatic family members have an increased incidence of neurological dysfunction and neuroimaging findings associated with MS (Xia et al., 2017). Additionally, patients with radiologically isolated syndrome (i.e., patients who meet MS criteria radiologically but are clinically asymptomatic) are known to be at high risk for development of clinical MS (Hosseiny et al., 2020). However, studies of asymptomatic individuals require large sample sizes, which cause recruitment and cost restraints. The reduced cost of low-field MRI could significantly impact the type of population based and longitudinal studies available to researchers by increasing sample sizes and allowing for more distributed recruitment outside academic medical centers (Deoni et al., 2022, Deoni et al., 2022). While making novel study designs (such as enrolling disabled patient populations) feasible, there are significant challenges, including lower resolution and reduced sensitivity, which must be addressed when planning such studies.

While machine learning methods for MS lesion segmentation have yet to consistently outperform manual segmentation, they reduce the cost, time, and subjectivity associated with manual labeling (Valcarcel et al., 2018, Valcarcel et al., 2018). Combining low-field MRI with automated techniques can further address barriers to MRI access and image interpretation. Additionally, automated segmentation could serve as a biomarker for determining eligibility or endpoints in clinical trials or as a starting point for further manual refinements. In our work, the average Dice overlap between automated 3T and 64mT segmentations was only 0.23, with three subjects having no overlap. The low overlap was driven in part by peripheral artifacts and flow-related venous hyperintensities on 64mT FLAIR imaging, which also resulted in a higher number of false positives (22% for lesions > 1.5 ml) despite comparatively high lesion sensitivity (100% for lesion > 1.5 ml). Pulse sequences or reconstruction could be further optimized to remove artifacts and increase resolution. The 64mT system uses a transmit/receive head coil and 3D acquisitions without modifications to reduce flowing spins. More practically, high-field lesion detection algorithms can be retrained or tuned to address differences in image quality and tissue contrast between field strengths.

The current study has several limitations. Our findings suggest that portable 64mT FLAIR scans are sensitive for white matter lesions in MS and more generally, but we focused on patients with established MS and did not assess the specificity of MS lesion detection relative to other disease processes or normal controls. In addition, sensitivity at the patient or lesion level will depend on the lesion volume and size distribution (Commowick et al., 2018). We used automated 3T segmentation as ground truth, though complete labeling accuracy is challenging even at high field, and we considered lesion overlap and volume rather than lesion counts. We only evaluated a single lesion segmentation method, and results may not generalize to other algorithms. Indeed, our findings indicate that both image acquisition strategies and segmentation methods can be further optimized to increase the sensitivity and accuracy of low field lesion detection for larger prospective studies. More accurate SNR measurements require multiple acquisitions (Dietrich et al., 2007), which were not available in this study design. We did not assess longitudinal imaging or the ability to detect new or active lesions. Gadolinium was only administered at one of the two sites and was not administered directly for 64mT imaging. As discussed above, given that none of the patients in our cohort had gadolinium-enhancing lesions on their high-field scans and the post-contrast delay before each 64mT scan, we cannot assess whether contrast enhancing lesions can be seen at 64mT.

## 5 Conclusion

In conclusion, increased imaging capabilities and potential portability of low-field MRI systems warrants their re-evaluation across a range of pathologies and indications. We found that a portable 64mT scanner was sensitive to brain WMLs in MS patients and that an automated algorithm designed for 3T image segmentation could be applied to the 64mT data. Although additional work will be needed to evaluate portable low-field MRI systems and their capacity to carry out specific clinical functions, our findings suggest promising avenues to more accessible imaging technologies for MS around the world.

### CRediT authorship contribution statement

**T. Campbell Arnold:** Conceptualization, Data curation, Methodology, Software, Formal analysis, Writing – original draft, Writing – review & editing. **Danni Tu:** Data curation, Methodology, Formal analysis, Writing – original draft, Writing – review & editing. **Serhat V. Okar:** Conceptualization, Data curation, Methodology, Formal analysis, Writing – original draft, Writing – review & editing. **Govind Nair:** Conceptualization, Methodology, Writing – review & editing. **Samantha By:** Conceptualization, Methodology, Writing – review & editing. **Karan D. Kawatra:** Data curation, Writing – review & editing. **Timothy E. Robert-Fitzgerald:** Data curation, Formal analysis, Writing – review & editing, Supervision, Funding acquisition. **Lisa M. Desiderio:** Data curation, Writing – review & editing, Supervision, Project administration, Funding acquisition. **Matthew K. Schindler:** Methodology, Writing – review & editing. **Russell T. Shinohara:** Conceptualization, Methodology, Writing – review & editing, Supervision, Funding acquisition. **Daniel S. Reich:** Conceptualization, Methodology, Formal analysis, Writing – review & editing, Supervision, Funding acquisition. **Joel M. Stein:** Conceptualization, Methodology, Formal analysis, Writing – review & editing, Supervision, Funding acquisition.

## Declaration of Competing Interest

The authors declare the following financial interests/personal relationships which may be considered as potential competing interests: Joel M. Stein reports financial support was provided by Hyperfine. Daniel S. Reich reports financial support was provided by Abata Therapeutics. Daniel S. Reich reports financial support was provided by Sanofi Genzyme. Daniel S. Reich reports financial support was provided by Vertex Pharmaceuticals. Samantha By reports a relationship with Hyperfine that includes: employment. Samantha By reports a relationship with Bristol Myers Squibb Co that includes: employment. Russell T. Shinohara reports a relationship with Octave Bioscience that includes: consulting or advisory. Russell T. Shinohara reports a relationship with American Medical Association that includes: consulting or advisory. Joel M. Stein reports a relationship with Centaur Diagnostics that includes: consulting or advisory.
