# Supplementary material for: Sensitivity of portable low-field magnetic resonance imaging for multiple sclerosis lesions
Source: Neuroimage Clin. 2022 Jun 27;35:103101. doi: 10.1016/j.nicl.2022.103101 (PMC9421456; doi:10.1016/j.nicl.2022.103101)
Supplement: Supplementary data 1 [file mmc1.docx]

**Supplemental Material**

*Study size calculation*

Based on preliminary results, the maximum diameter (Dmax) of the smallest lesion on 3T imaging was estimated to be 2 mm, while Dmax was estimated to be 6 mm on 64mT imaging. The standard deviation for manual measurements was estimated to be 2 mm. Using a significance level of 0.01 and a power level of 0.95, we estimated the necessary sample size to be 18 patients to detect a significant difference between modalities (Eng 2003). Our study is well powered with over 30 patients.

1. Eng J. Sample size estimation: how many individuals should be studied? Radiology. 2003 May;227(2):309-13. Doi: 10.1148/radiol.2272012051. PMID: 12732691.


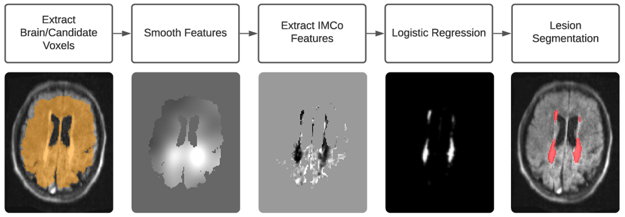


**Figure S1. Automated segmentation algorithm.** Visual description of the steps in the MIMoSA algorithm.


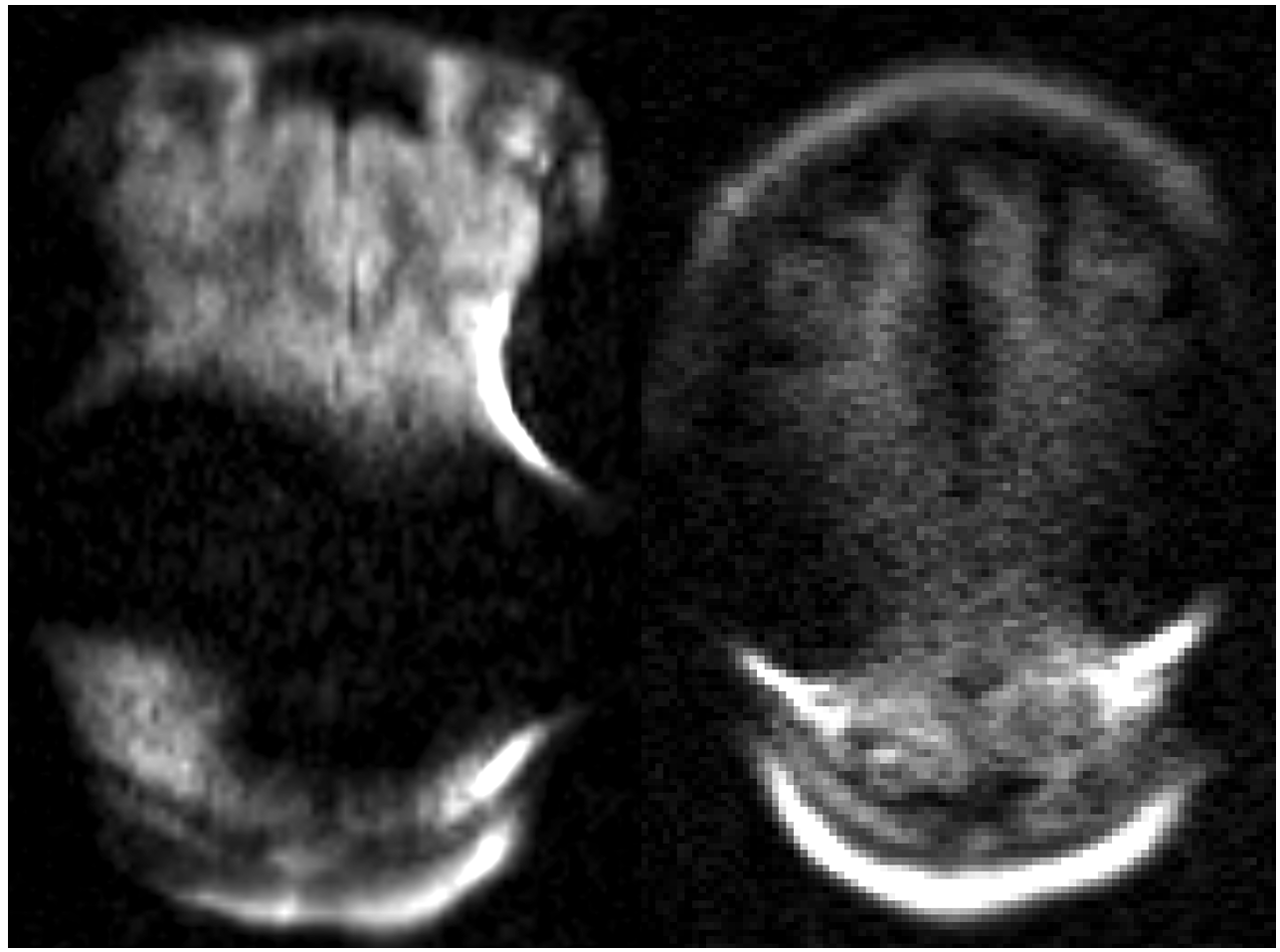


**Figure S2. 64mT image artifacts.** One subject was excluded due to significant image artifacts at 64mT. The artifacts appeared bilaterally and were present on both FLAIR (left) and T1w (right) imaging. The T1w sequence was repeated several times and artifacts persisted throughout the acquisitions; after this the 64mT protocol for this patient was terminated. The patient was not wearing jewelry and did not have metal implants. We believe the artifacts may have been caused by a nearby large metal box, used to house the portable MRI when not in use.


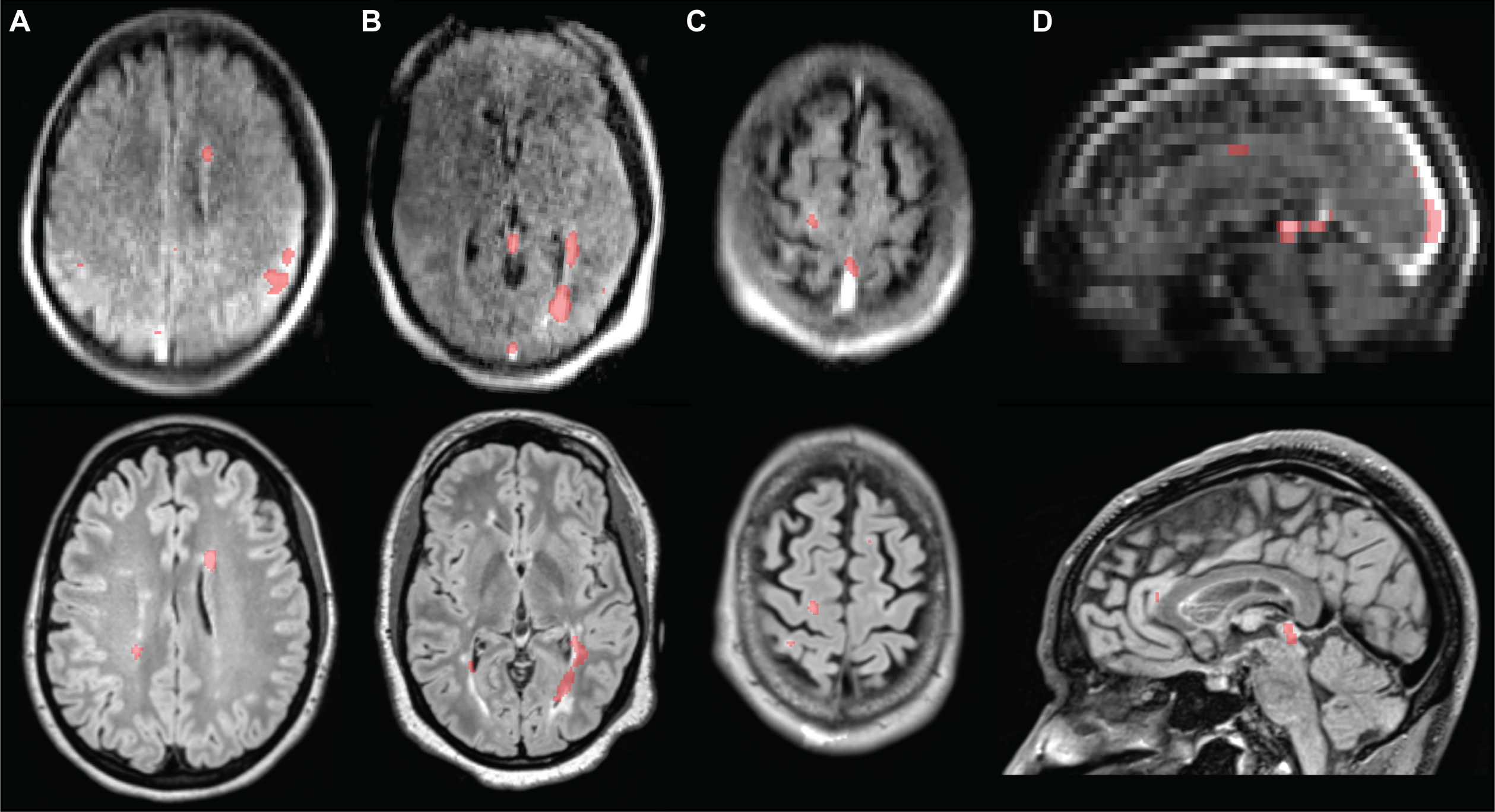


**Figure S3. False positive lesion segmentations on 64mT T2-FLAIR.** This figure illustrates common causes of 64mT false positive lesion detections on the top row with 3T comparisons provided on the bottom row. False positive detections were primarily caused by artifactual peripheral hyperintensity, as seen in (A) a 33-year-old female with RRMS, and hyperintense venous structures, as seen in (B) a 35-year-old female with RRMS, (C) a 55-year-old male with RRMS, and (D) a 50-year-old male with clinically isolated syndrome. Other hyperintense structures, such as a pineal cyst in panel D, were also a source of false positive labeling in both 3T and 64mT images.


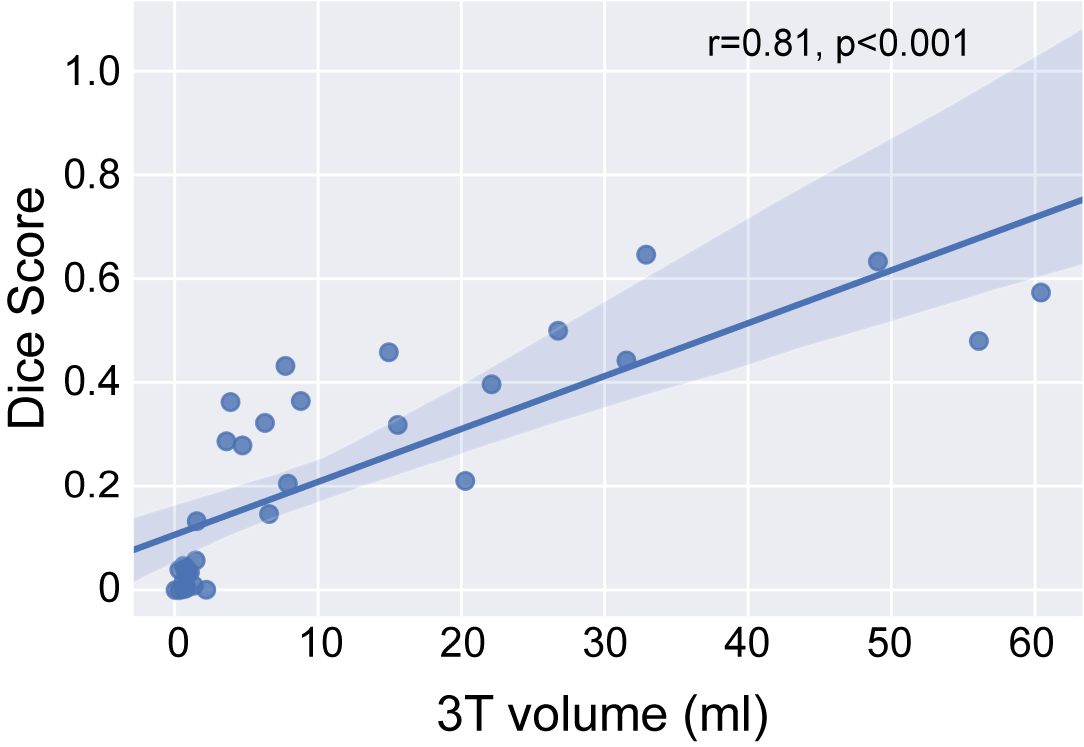


**Figure S4. Dice score increases with lesion volume.** Previous studies have found that larger lesion sizes are associated with higher Dice scores (34). In our study, we found a similar effect (r = 0.81, p < 0.001) such that subjects with higher lesion volume had correspondingly higher Dice scores.


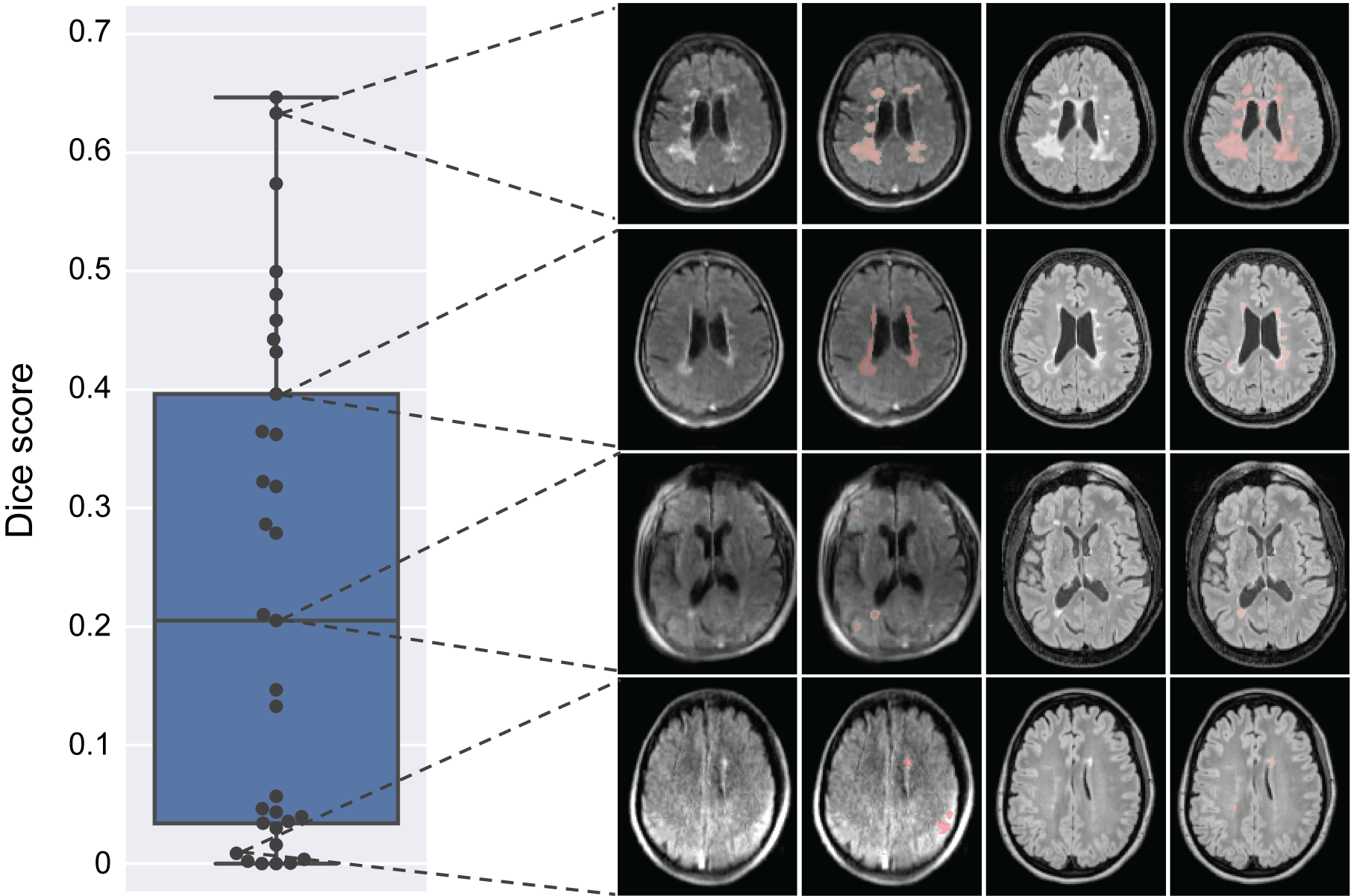
**Figure S5. Dice score distribution of lesion segmentations.** This figure highlights patients from each quartile of the Dice score distribution (mean = 0.23, standard deviation = 0.21). From left to right, the images are the original 64mT image, 64mT image with segmentation overlay, the original 3T image, and 3T image with segmentation overlay. Even patients in the lowest quartile demonstrate overlapping periventricular lesions.

*False positive detections*

Visual inspection of automated 64mT segmentations revealed some consistent sources of false positive lesion detection on T2-FLAIR images, including likely flow-related hyperintensities in venous structures (dural venous sinuses and small cerebral or cortical veins), other hyperintense structures such as pineal cysts, and peripheral hyperintensities due to image artifacts. Examples of these artifacts can be seen in figure S3.

In a sub-analysis, automated segmentations were manually edited in ITK-SNAP to remove some of the common false positives. In particular, we removed false positives in major venous sinuses (i.e., sagittal, straight, and transverse) as well as hyperintense pineal cysts. Peripheral hyperintensities caused by image artifacts or smaller cortical veins were not edited, as classification of these detections is more subjective. After manually editing the segmentations, the true positive rate (TPR) and false discovery rate (FDR) were recalculated.

There was a sharp decrease in the FDR as a function of lesion size after manual editing, reaching 11% at >1.0 ml compared to 36% in the original automated segmentation (Fig. S6A). There was a modest decrease in FDR as a function of lesion intensity; however, FDR remains >65% across sensitivities (>75% in automated segmentations) (Fig. S6B). TPR remains relatively unchanged in both plots.


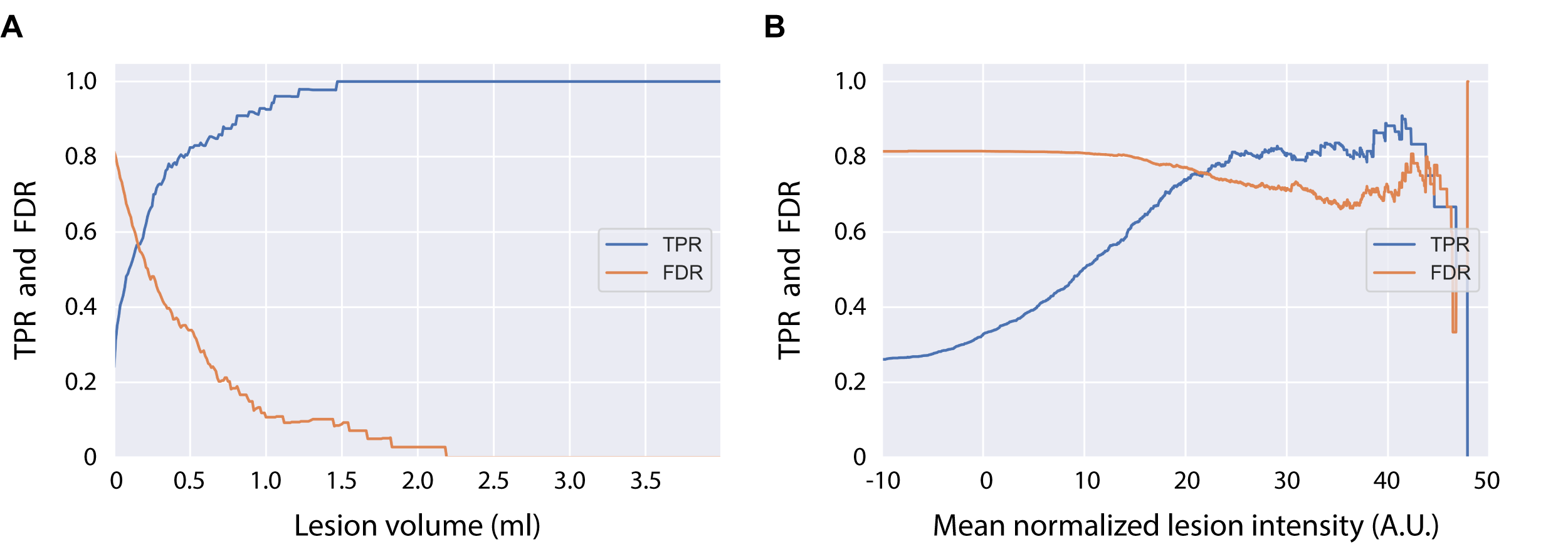


**Figure S6. False discovery rate decreases after removing hyperintense veins.** After manually removing hyperintense veins and other hyperintense brain structures from segmentations, the false discovery rate (FDR) decreases. (A) The FDR decreases as a function of lesion size. Manually edited segmentations had a lower FDR relative to the unedited automated segmentations (>1 ml: unedited=36%, edited=11%, >1.5ml: unedited=22%, edited=9%, >2.5ml: unedited=3%, edited=0%). (B) When analyzing FDR as a function of mean lesion intensity, the effect was less pronounced. In unedited segmentations, FDR was high (>75%) across lesion intensities. While there was a modest reduction after manual editing, FDR remained >65%.


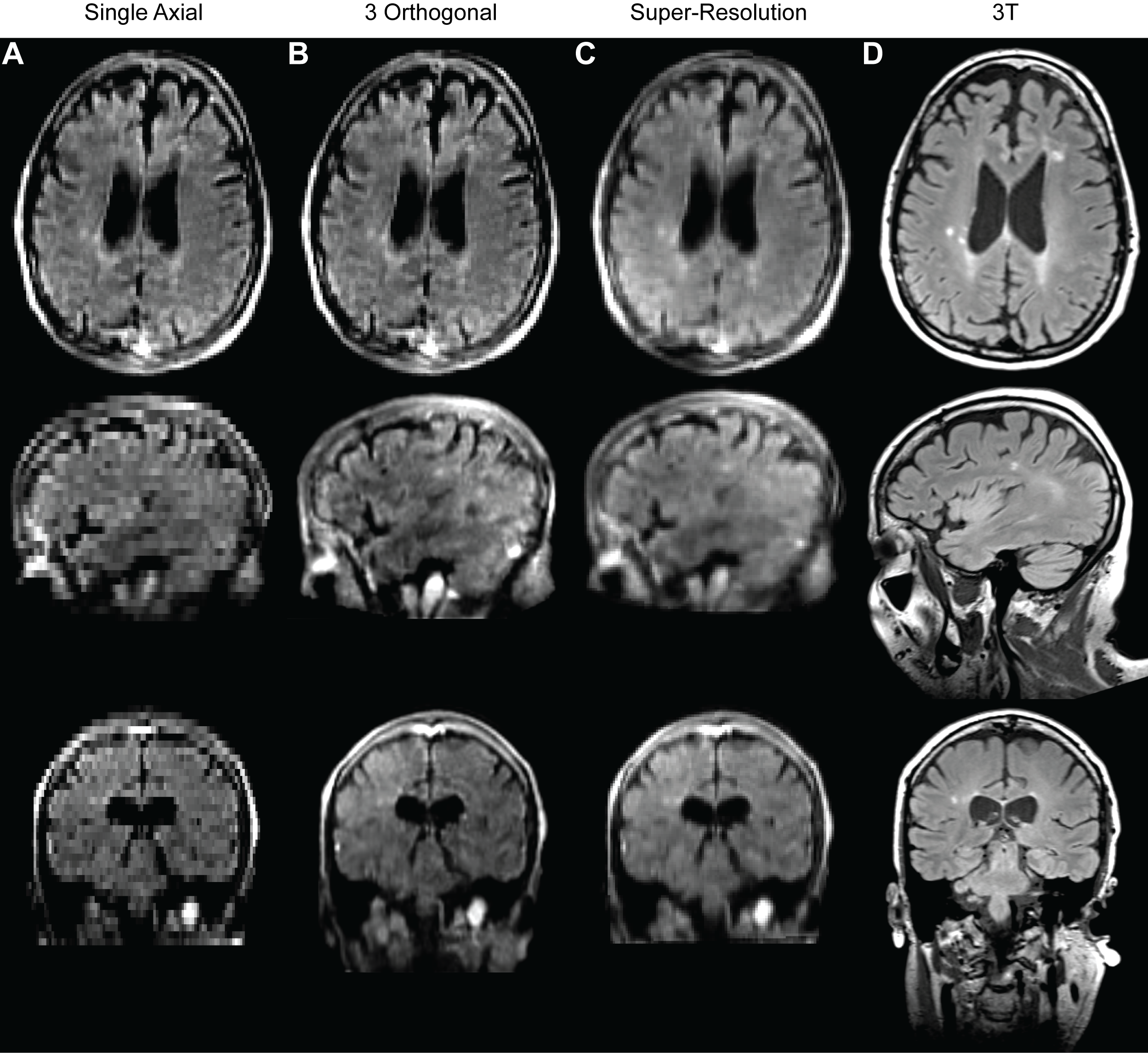


**Figure S7. FLAIR super-resolution image generated from a control participant with incidental nonspecific white matter lesions**. (A) A single 64mT axial T2-FLAIR (scan time = 6:03 min). (B) Three orthogonal 64mT T2-FLAIR acquisitions coregistered to the axial sequence (scan time: sagittal = 5:02 min, coronal = 6:02 min). (C) 64mT T2-FLAIR 1.8 mm isotropic super-resolution image generated from the three orthogonal views. (D) Comparison 3T T2-FLAIR image collected 15 months prior. Full sequence parameters are available in supplementary table S1.


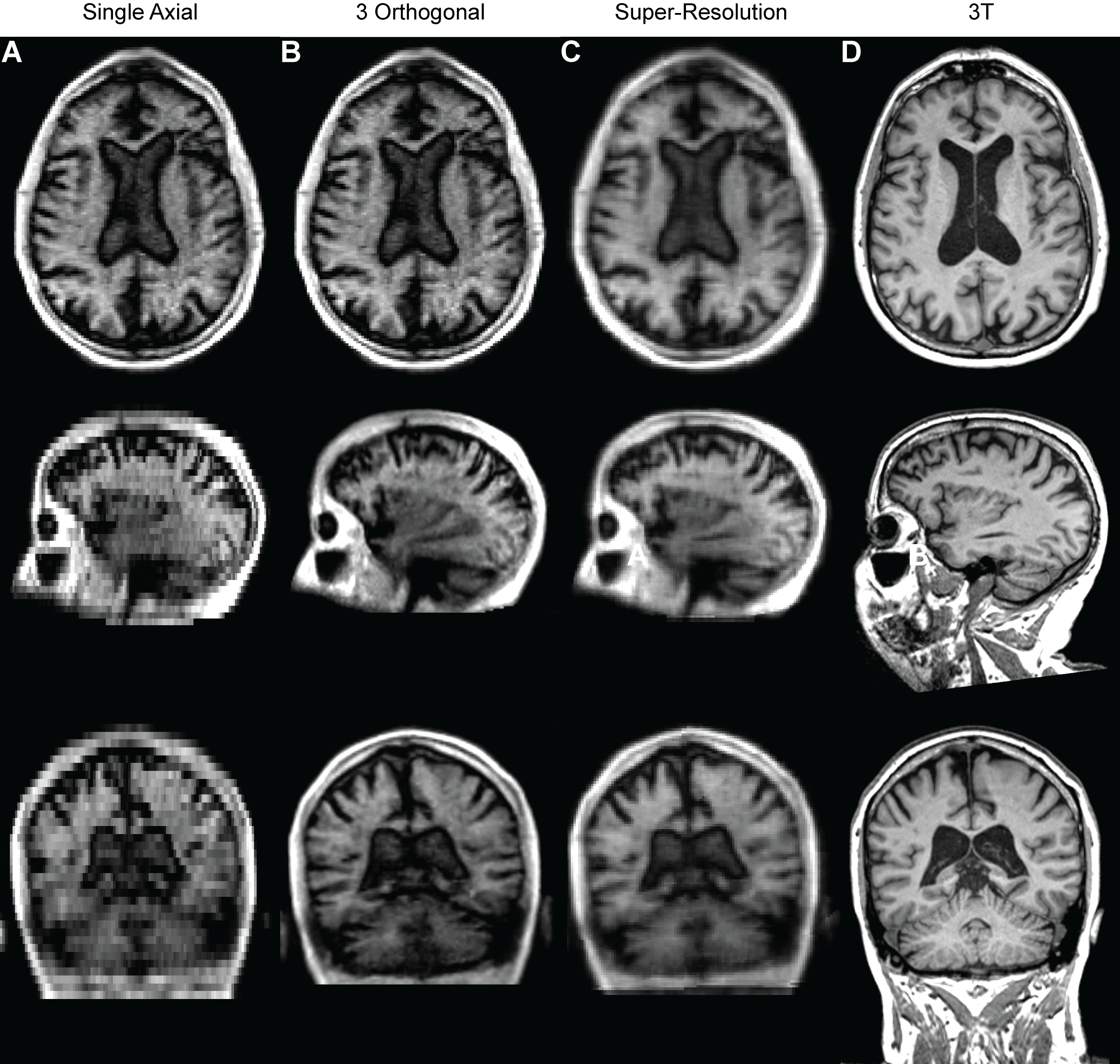


**Figure S8. T1w super-resolution image generated from a control participant with incidental nonspecific white matter lesions**. (A) A single 64mT axial T1w (scan time = 5:39 min). (B) Three orthogonal 64mT T1w acquisitions coregistered to the axial sequence (scan time: sagittal = 5:39 min, coronal = 5:32 min). (C) 64mT T1w 1.6 mm isotropic super-resolution image generated from the three orthogonal views. (D) Comparison 3T T1w image collected 15 months prior. Full sequence parameters are available in supplementary table S1.

**
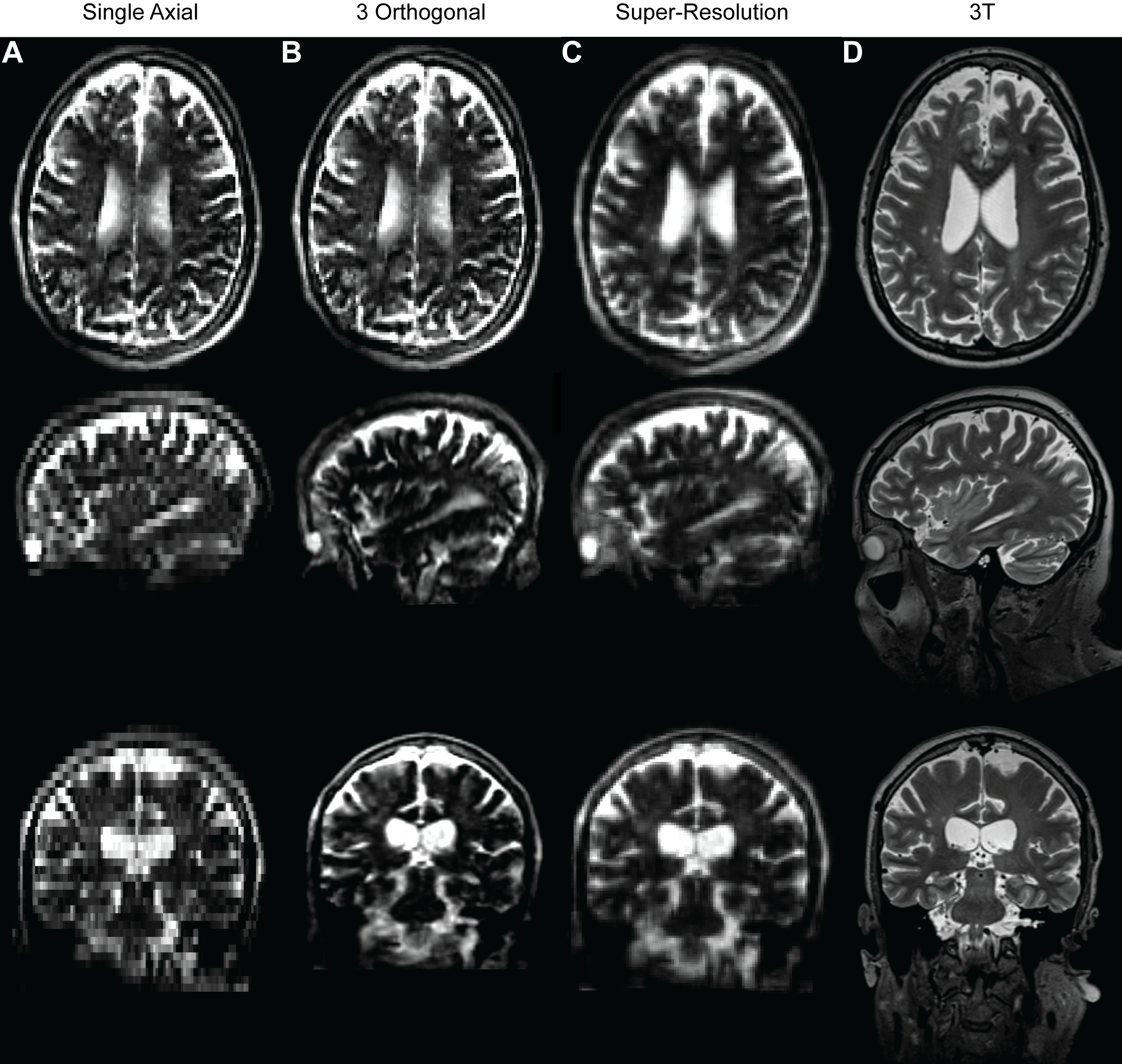
**

**Figure S9. T2w super-resolution image generated from a control participant with incidental nonspecific white matter lesions.** (A) A single 64mT axial T2w (scan time = 2:53 min). (B) Three orthogonal 64mT T2w acquisitions coregistered to the axial sequence (scan time: sagittal = 1:59 min, coronal = 2:21 min). (C) 64mT T2w 1.6 mm isotropic super-resolution image generated from the three orthogonal views. (D) Comparison 3T T2w image collected 15 months prior. Full sequence parameters are available in supplementary table S1.

| Sequence | Plane | Field Strength (T) | TE (ms) | TR (s) | TI (s) | Resolution (mm) | Scan-time (min:sec) | Averages |
| --- | --- | --- | --- | --- | --- | --- | --- | --- |
| T1w | axial | 0.064 | 5.96 | 1.5 | 0.3 | 1.6x1.6x5.0 | 5:39 | 2 |
|  | sagittal | 0.064 | 5.88 | 1.5 | 0.3 | 5.0x1.6x1.6 | 5:39 | 2 |
|  | coronal | 0.064 | 5.59 | 1.5 | 0.3 | 1.6x5.0x1.6 | 5:32 | 2 |
|  | isotropic | 3 | 2.52 | 1.9 | 0.9 | 1.0x1.0x1.0 | 3:10 | 1 |
| T2w | axial | 0.064 | 182.4 | 2 | N/A | 1.6x1.6x5.0 | 2:53 | 4 |
|  | sagittal | 0.064 | 230 | 2 | N/A | 5.0x1.6x1.6 | 1:59 | 4 |
|  | coronal | 0.064 | 217.6 | 2 | N/A | 1.6x5.0x1.6 | 2:21 | 4 |
|  | isotropic | 3 | 408 | 3.2 | N/A | 1.0x1.0x1.0 | 5:57 | 1 |
| T2-FLAIR | axial | 0.064 | 183.6 | 4 | 1.4 | 1.8x1.8x5.0 | 6:03 | 4 |
|  | sagittal | 0.064 | 234.4 | 4 | 1.4 | 5.0x1.8x1.8 | 5:02 | 4 |
|  | coronal | 0.064 | 217.2 | 4 | 1.4 | 1.8x5.0x1.8 | 6:02 | 4 |
|  | isotropic | 3 | 289 | 6 | 2.2 | 1.0x1.0x1.0 | 4:42 | 1 |

**Table S1. Sequence parameters for generating 64mT super-resolution images and 3T comparison.** Abbreviations: Tesla (T), T1-weighted (T1w), T2-weighted (T2w), Fluid-attenuated inversion recovery (FLAIR), echo time (TE), repetition time (TR), inversion time (TI).
